# New prognostic features and personalized treatment strategies of mitochondrial related genes in colorectal cancer patients

**DOI:** 10.3389/fphar.2025.1540767

**Published:** 2025-04-01

**Authors:** Qizheng Xu, Zhiwen Wang, Shan-Tao Huang, Jia-Yu Shi, Yan Zhu, Han-Qing Pang

**Affiliations:** ^1^ School of Life Science, Shanxi University, Taiyuan, China; ^2^ Institute of Translational Medicine, School of Medicine, Yangzhou University, Yangzhou, China

**Keywords:** colorectal cancer, mitochondria, prognostic prediction, treatment effects, chemotherapeutic agents

## Abstract

Colorectal cancer (CRC) is a common and aggressive malignancy with the complex and varied molecular landscape. Mitochondria play a pivotal role in the metabolic reprogramming of cancer cells, and their function can profoundly influence tumor progression. Therefore, identifying mitochondrial genes with immune-related features may offer a promising new approach for prognosis in CRC. Mitochondrial-associated genes were retrieved from the MITOCARTA 3.0 dataset. The LASSO regression method was applied to identify prognostic genes, while the area under the ROC curve and nomograms were used to assess the robustness of the model. Single-sample genomic enrichment analysis (ssGSEA) was utilized to explore the relationship between model genes and immune infiltration, and drug sensitivity analysis was conducted to identify potential therapeutic agents. Cellular assays were performed to validate the effectiveness of identified drugs. Key mitochondrial genes, including SUCLG2, ACACB, OSBPL1A, and TRAP1, have been identified as significant prognostic markers in CRC. The expression of ACACB and OSBPL1A progressively increased, while SUCLG2 and TRAP1 expression decreased in patients. ROC curve analysis of the TCGA dataset showed an area under the curve (AUC) greater than 0.6 for 1-, 2-, and 3-year survival predictions, demonstrating the strong prognostic potential of this model. Additionally, the model was strongly correlated with immune cells, particularly CD8^+^ T cells, and immune checkpoint regulators. Molecular docking analysis revealed that OSBPL1A binds to dabrafenib at glycine position 747. Cellular assays confirmed that dabrafenib effectively inhibited CRC cell migration and proliferation, providing a promising therapeutic avenue. Our findings suggested that the four mitochondrial-related genes identified in this study provide accurate survival predictions for CRC patients.

## 1 Introduction

Colorectal cancer (CRC) is the most prevalent malignant tumor in the gastrointestinal tract. It ranks as the third most diagnosed cancer and the second leading cause of cancer-related deaths globally, with approximately 1.9 million new cases annually (Sung et al., 2021; Armaghany et al., 2012; Tariq and Ghias, 2016). CRC is classified into stages from I to IV based on tumor growth and metastasis. Later stages are generally associated to significantly lower survival rates (An et al., 2024). CRC has a favorable prognosis when diagnosed early; however, 15%–30% of patients already present with metastatic disease at diagnosis (Moisuc et al., 2024). Early diagnosis and accurate risk stratification play a crucial role in improving CRC treatment outcomes, with colonoscopy and imaging techniques, such as computed tomography (CT) and magnetic resonance imaging (MRI), serving as the standard diagnostic methods (Milot et al., 2024). However, these diagnostic approaches sometimes fail to differentiate between tumor recurrence and benign lesions, which can lead to treatment delays (Rompianesi et al., 2022). Standard therapeutic strategies for CRC include surgical resection, chemotherapy, and radiotherapy. Nevertheless, resistance to these treatments, along with frequent tumor recurrence and metastasis, makes CRC treatment particularly challenging (Kumar et al., 2023). Hence, developing innovative prognostic models and identifying effective drug targets are essential for improving early diagnosis and treatment outcomes for CRC.

Mitochondria, known as the cellular powerhouses, are crucial for bioenergetics, metabolic signaling, and cell survival regulation. Its functions significantly influence tumor progression and resistance to therapy (Roy et al., 2024). Emerging evidence indicates that mitochondrial dysfunction and metabolic reprogramming play a role in developing chemotherapy resistance in CRC (Abdelmaksoud et al., 2023). Tumor cells often prefer aerobic glycolysis, known as the Warburg effect, which enables them to grow continuously under low oxygen conditions while still producing ATP (Flood et al., 2023). Furthermore, CRC cells heavily depend on mitochondrial oxidative phosphorylation (OXPHOS) for ATP production, especially when glucose is scarce (Cherkas et al., 2020). This ability to switch metabolic pathways enables tumor cells to adapt to different microenvironmental conditions, promoting their growth and metastasis. Nevertheless, the influence of genes associated with mitochondrial function on the prognosis and response to therapy in CRC is still not well understood.

This study investigated mitochondrial-related differentially expressed genes (Mit-DEGs) in CRC by analyzing publicly available mRNA expression profiles. We constructed prognostic models based on mitochondrial-associated genes using the TCGA dataset, which were then validated with the GEO and other independent datasets. Additionally, we examined protein expression levels of model-associated genes in normal colon and CRC tissues, and performed functional annotations of risk groups to uncover potential mechanisms. Finally, we explored mitochondrial-related therapeutic targets in colorectal cancer and showed that specific compounds could covalently bind to mitochondrial proteins, potentially inhibiting the proliferation and invasion of CRC cells.

## 2 Materials and methods

### 2.1 Data acquisition

RNA-seq data for 634 CRC samples and 51 normal colorectal tissue samples were obtained from the UCSC Xena platform (http://xena.ucsc.edu/) and The Cancer Genome Atlas (TCGA) database (https://portal.gdc.cancer.gov/), along with corresponding clinical information. A total of 1136 mitochondrial-related genes were retrieved from the MitoCarta3.0 database (https://www.broadinstitute.org/mitocarta) to assess their expression in CRC tissue. Detailed descriptions of the datasets are provided in Table 1.

**TABLE 1:** Basic information of datasets used in the study.

GSE series	Tissue	Organism	Sample size		Platform
			Normal	Tumor	
COAD	Colon	*Homo sapiens*	41	471	TCGA
READ	Rectum	*Homo sapiens*	10	163	TCGA
Mitochondria-related genes	—	*Homo sapiens*	—	—	MitoCarta3.0
GSE20916	CRC	*Homo sapiens*	34	30	GEO
GSE21510	CRC	*Homo sapiens*	25	123	GEO

### 2.2 Identification of differentially expressed genes

The RNA-seq data from CRC samples and normal colorectal tissues were combined and subjected to analysis using R software (version 4.2.1). Differentially expressed genes (DEGs) were identified based on the criteria of |log2FoldChange| ≥ 1 and p-value <0.05, using the “limma” and “edgeR” R packages. The DEGs were then intersected with mitochondrial-related genes to identify Mit-DEGs in CRC.

### 2.3 Construction and validation of prognostic model

The Mit-DEGs were further analyzed to construct a prognostic model (Xiao et al., 2022; Zhang et al., 2024). First, univariate Cox regression analysis was performed on the training dataset to identify genes significantly associated with overall survival (OS) in patients. These candidate genes were refined using LASSO Cox regression analysis with the “glmnet” R package. The performance of the prognostic model was evaluated by calculating risk scores for patients in both the training and validation datasets. Kaplan-Meier survival analysis was conducted using the “survival” and “survminer” packages. Model accuracy was assessed with time-dependent ROC curves, and the area under the curve (AUC) was calculated for 1-, 2-, and 3-year survival predictions.

### 2.4 Nomogram construction and ROC curves

A nomogram was developed based on significant independent prognostic factors identified through multivariate Cox regression analysis. The predictive accuracy and clinical utility of the nomogram were assessed using calibration plots for 1-, 2-, and 3-year OS. The C-index was calculated to assess the model’s discriminative ability, and ROC curves were generated to compare predicted survival with actual survival outcomes.

### 2.5 Immune infiltration analysis

We analyzed the immune landscape of CRC using ssGSEA. Using the CIBERSORT algorithm, we calculated various immune-related scores, such as immune cell abundance, immune score, stromal score, and tumor purity. Correlation analysis was performed to evaluate the relationship between immune cell infiltration and the risk scores generated by the prognostic model.

### 2.6 Drug screening and molecular docking

We identified potential small-molecule drugs targeting the key DEGs were identified using the CellMiner database (https://discover.nci.nih.gov/cellminer/home.do) and DrugBank database (https://www.drugbank.ca/). Molecular docking studies were conducted using AutoDock Vina to evaluate the binding affinity of candidate compounds with key proteins encoded by the DEGs. PyMOL (v.2.4.0) was used for visualization and analysis of docking results (Pang et al., 2018).

### 2.7 Cell culture and reagents

Human CRC cell lines HCT116 and SW480 were obtained from the American Type Culture Collection (ATCC). Cells were maintained in RPMI-1640 medium supplemented with 10% fetal bovine serum (FBS) and 1% penicillin-streptomycin, cultured in a 5% CO_2_ incubator at 37°C. The overexpressed OSBPL1A plasmid was purchased from Miaoling Biotechnology Co., Ltd. (China, Wuhan). Using X-tremeGENE according to the manufacturer’s specifications, HP DNA Transaction Agent (Roche# 06366236001) transfected the plasmid into cells.

### 2.8 Cell proliferation and migration detection

Evaluate the proliferation rate of CRC cells using plate cloning technology. As described in the previous reference to CRC cell research methods, colony formation and pore migration experiments were conducted.

### 2.9 Statistical analysis

Statistical analysis was conducted using R software and GraphPad Prism (v. 8.0). Student’s t-test and Wilcoxon rank-sum test were used as appropriate. A p-value < 0.05 was considered statistically significant, and results were presented as mean ± standard deviation (SD).

## 3 Result

### 3.1 Identification of Shared Pathogenic Genes in CRC

**FIGURE 1:**
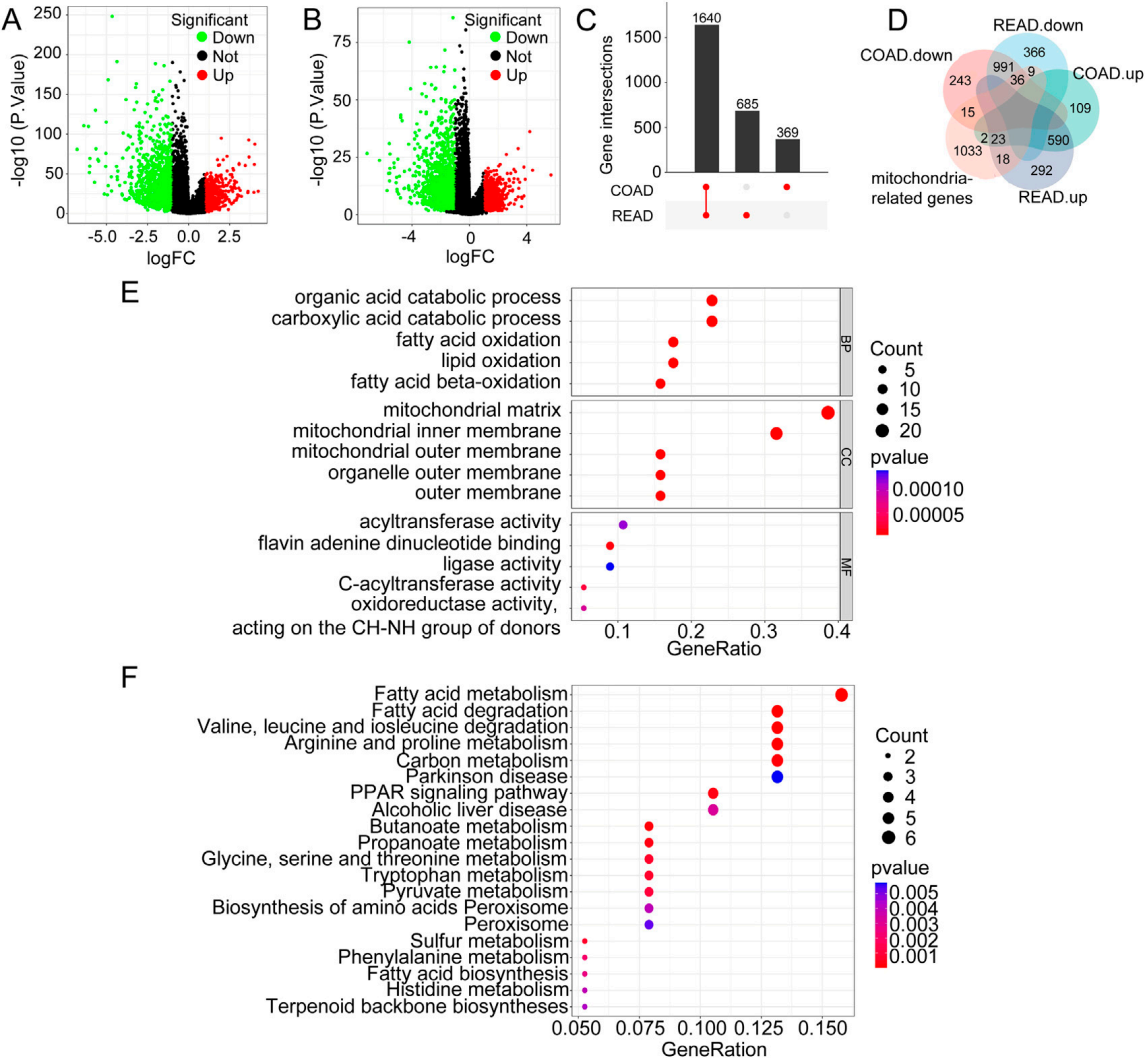
Identification of Shared Pathogenic Genes in CRC. **(A)** Volcano plot illustrating the DEGs in COAD. **(B)** Volcano plot of DEGs in READ. **(C)** UpSet plot showing the overlap of DEGs between COAD and READ. **(D)** Venn diagram of MRGs and their association with DEGs in COAD and READ. **(E)** GO enrichment analysis of Mit-DEGs. **(F)** KEGG enrichment analysis of Mit-DEGs.

Figure 1A displaysa volcano plot of DEGs in COAD, highlighting significantly upregulated and downregulated genes based on log_2_FC and p-values. Figure 1B presents a similar volcano plot for DEGs in READ, emphasizing genes with significant expression changes. The UpSetR plot in Figure 1C visualizes the overlap of DEGs between COAD and READ, showing that 1,640 genes are differentially expressed only in COAD, 685 genes are commonly differentially expressed in both COAD and READ, and 369 genes are differentially expressed only in READ. These findings indicate that although specific gene sets exhibited unique expression patterns in each condition, there was also a significant overlap of DEGs between COAD and READ.

Furthermore, the MRGs, comprising approximately 1,136 genes, were compared with the DE mRNA data from COAD and READ. Figure 1D displays a Venn diagram showing the overlap of upregulated and downregulated MRGs between COAD and READ, identifying 59 common genes—36 downregulated and 23 upregulated. To better understand the functional roles of the 59 Mit-DEGs, we conducted GO and KEGG pathway enrichment analyses. GO analysis showed significant enrichment in processes including organic acid catabolism, fatty acid oxidation, and components of the mitochondrial inner membrane. This highlights the role of mitochondria in energy metabolism and redox activities (Figure 1E). Additionally, the KEGG pathway analysis identified pathways related to fatty acid metabolism, carbon metabolism, and other metabolic processes, emphasizing the critical role of mitochondrial metabolism in CRC (Figure 1F). The GSEA enrichment analysis indicated that the Mit-DEGs mainly exert their influence on mitochondrial function via the pathways including KEGG Oxidative Phosphorylation, KEGG Citrate Cycle TCA Cycle, KEGG Glycolysis Gluconeogenesis, KEGG Fructose and Mannose Metabolism, KEGG Drug Metabolism - Cytochrome P450, and KEGG Glutathione Metabolism (Supplementary Figure 1).

### 3.2 Interaction analysis of Mit-DEGs

**FIGURE 2:**
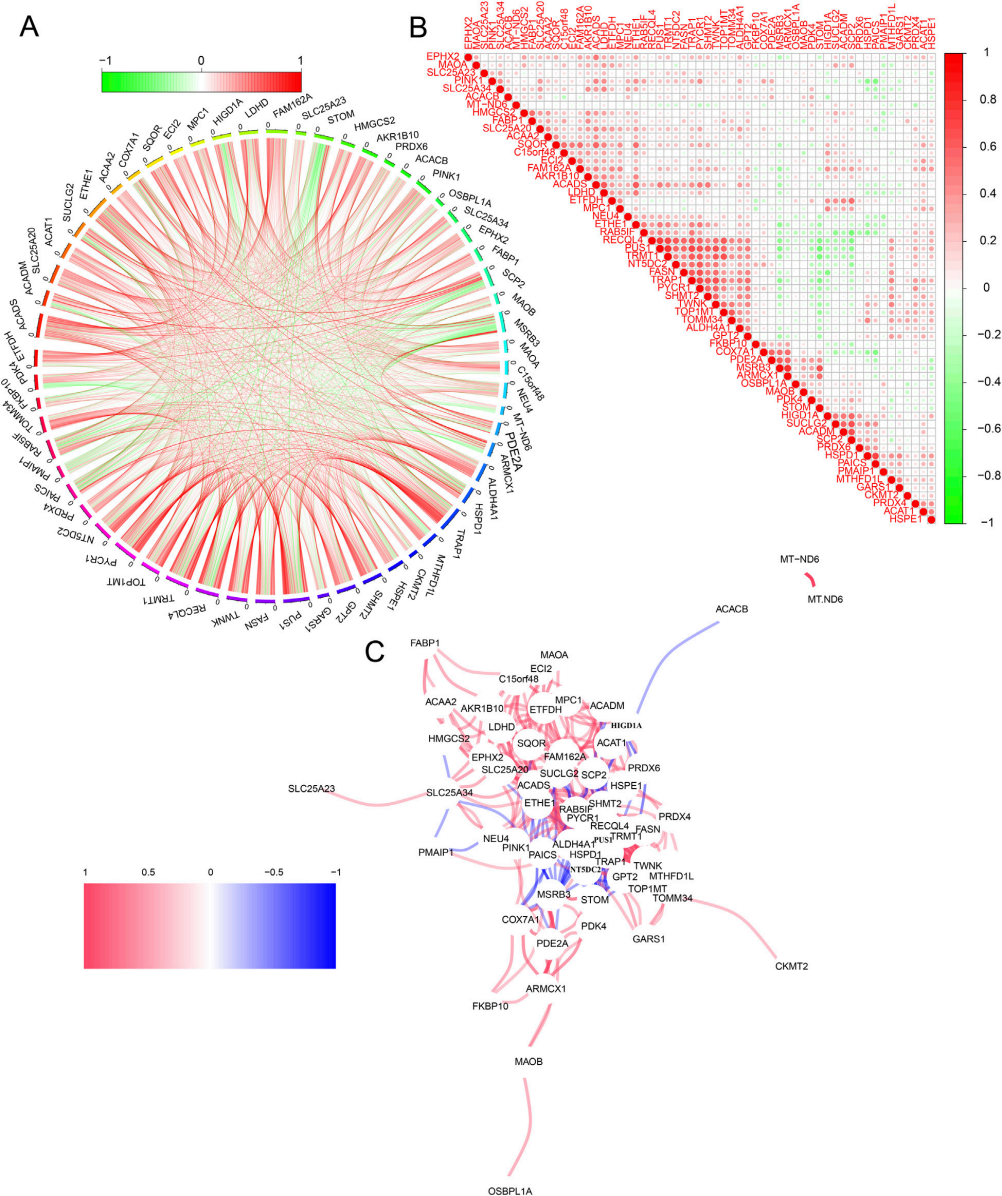
Interaction Analysis of Mit-DEGs. **(A)** Circular plot of Mit-DEG interactions. **(B)** Correlation heatmap of Mit-DEGs. **(C)** Network diagram of Mit-DEG interactions.

We conducted further analysis to explore the complex interaction network of the 59 overlapping genes that are either upregulated or downregulated in COAD and READ. The circular plot illustrates the interactions between genes. In this plot, red lines indicate positive correlations, while green lines indicate negative correlations (Figure 2A). The correlation heatmap quantifies the relationships between these genes, using a color scale that transitions from green (indicating negative correlation) to red (indicating positive correlation) (Figure 2B). Additionally, the network diagram illustrates gene interactions. In this diagram, nodes represent different genes, while the color and thickness of lines indicate the direction and strength of interactions. Red lines represent strong positive correlations and blue lines represent negative correlations (Figure 2C). Collectively, the circular plot, correlation heatmap, and network diagram indicated that the 59 overlapping genes in COAD and READ primarily function synergistically.

### 3.3 Construction of the mitochondrial-associated differential gene prognostic model

**FIGURE 3:**
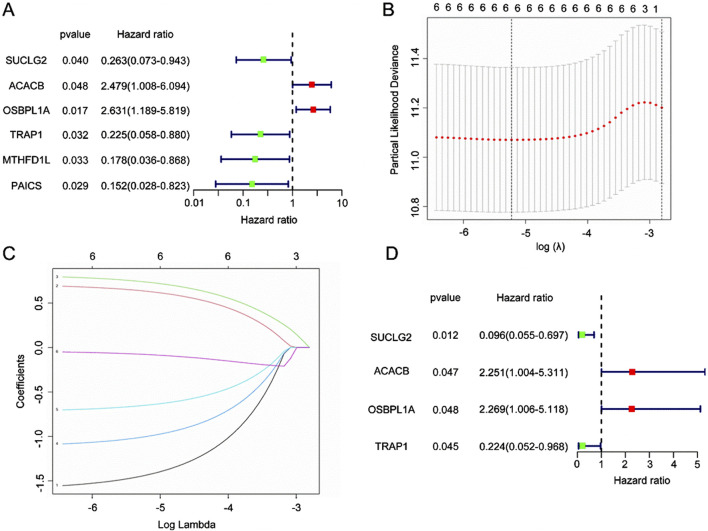
Construction of the Mitochondrial-Associated Differential Gene Prognostic Model. **(A)** Forest plot of univariate Cox regression analysis. Green squares represent low-risk genes (HR < 1), while red squares represent high-risk genes (HR > 1). **(B)** Cross-validation plot of LASSO regression. The optimal λ value is selected through cross-validation to minimize model bias, with the red dashed line indicating the position of the best λ. **(C)** Coefficient shrinkage path of LASSO regression. **(D)** Forest plot of multivariate Cox regression analysis.

The Mit-DEGs model is built using several statistical analyses, including univariate Cox regression, multivariate Cox regression, and Lasso regression. In Figure 3A, univariate Cox regression results indicated that ACACB and OSBPL1A (HR > 1) were high-risk genes, suggesting that higher expression levels are linked to poor patient prognosis. Conversely, SUCLG2, TRAP1, MTHFD1L, and PAICS were identified as low-risk genes because their higher expression levels were associated with better prognosis.

We applied LASSO regression to enhance gene selection and avoid overfitting. Figure 3B presents the cross-validation plot of LASSO, with the y-axis represents the partial likelihood bias and the x-axis corresponds to the logarithmic transformation of the regularization parameter λ. The optimal λ value, marked by the red dashed line, minimizes model bias. Figure 3C further illustrates the coefficient contraction path of LASSO, showing that as λ increases, the coefficients of non-informative genes decrease, thereby highlighting the most relevant genes for the prognostic model, including SUCLG2, ACACB, OSBPL1A, TRAP1, MTHFD1L, and PAICS.

Lastly, we performed multivariate Cox regression analysis to confirm the prognostic significance of the selected genes. The results (Figure 3D) confirm the prognostic value of four Mit-DEGs: ACACB, OSBPL1A, TRAP1, and SUCLG2. This comprehensive analysis identified key genes that influenced patient prognosis, offering a solid foundation for developing mitochondrial-related prognostic models.

### 3.4 Development of Mit-DEGs related functions

**FIGURE 4:**
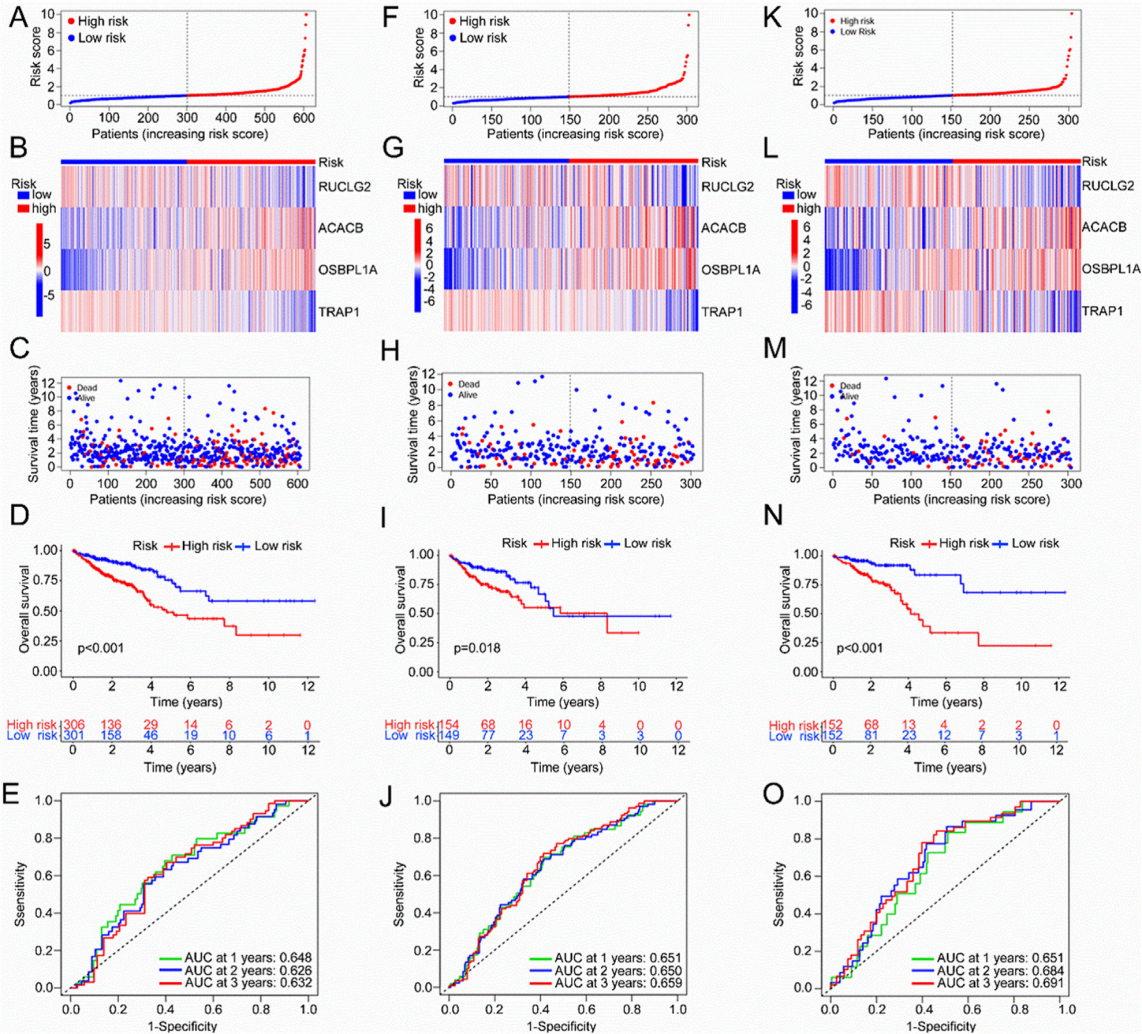
Validation of the prognostic model based on mitochondria-associated differential genes. **(A)** Expression levels of SUCLG2, ACACB, OSBPL1A, and TRAP1 in the TCGA cohort; **(B)** Scatter plot of the model in the TCGA cohort, classifying samples into high-risk and low-risk groups; **(C)** Scatter plot of the relationship between patient survival time and risk scores in TCGA samples; **(D)** Distribution of survival status among TCGA samples; **(E)** ROC curves for 1-, 2-, and 3-year survival in the TCGA cohort; **(F–J)** Validation of the prognostic model in the TCGA training set; **(K–O)** Validation of the prognostic model in the TCGA validation set.

The risk scores of 607 CRC patients were calculated, and the patients were classified into high-risk and low-risk groups based on the median risk score. In the scatter plot, patients are clearly separated into high-risk and low-risk groups (Figure 4A). ACACB and OSBPL1A exhibit high expression levels in the high-risk group, while SUCLG2 and TRAP1 are more highly expressed in the low-risk group (Figure 4B). The distribution of survival status for all TCGA samples is displayed (Figure 4C), indicating a poorer prognosis in the high-risk group compared to the low-risk group among TCGA CRC patients (Figure 4D, P < 0.001). The ROC curves for 1-, 2-, and 3-year survival prediction in all TCGA samples showed acceptable AUC values of 0.648, 0.626, and 0.632, respectively (Figure 4E). To further validate the model, the TCGA data were randomly divided into training and validation sets. The results in both the training and validation sets aligned with those observed in the entire dataset (Figures 4F–O). In the training set, survival analysis revealed a significantly lower survival rate for the high-risk group compared to the low-risk group (P < 0.018). The ROC curves for predicting 1-, 2-, and 3-year survival in all TCGA samples yielded AUC values of 0.648, 0.626, and 0.632, respectively (Figure 4E). Similarly, in the validation set, survival analysis demonstrated a significantly lower survival rate in the high-risk group (P < 0.001), with the ROC curves analysis showing AUC values of 0.651, 0.684, and 0.691 for 1-, 2-, and 3-year survival, respectively. Overall, the Mit-DEGs SUCLG2, ACACB, OSBPL1A, and TRAP1 are strong predictors of overall survival in CRC.

### 3.5 Exploration of key DEGs expression levels in GEO datesets

**FIGURE 5:**
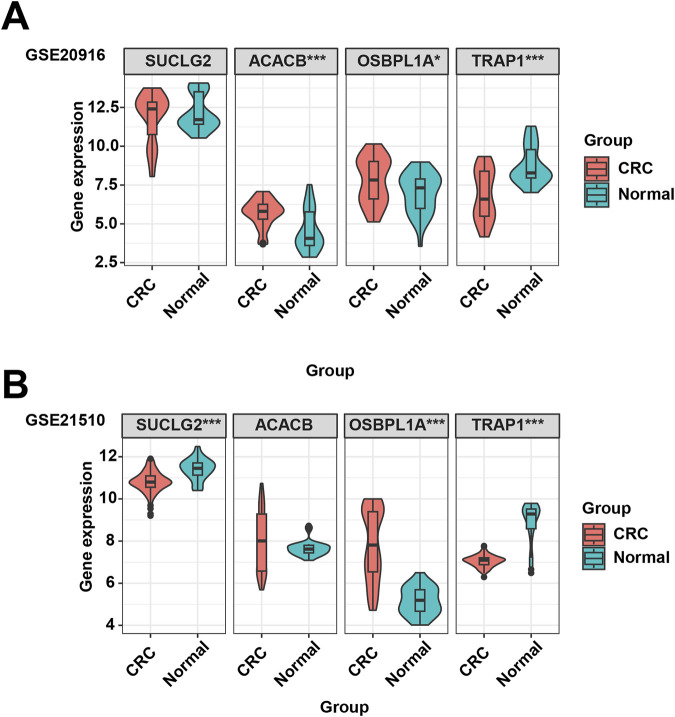
The GEO datasets were used to validate the expression of the SUCLG2, ACACB, OSBPL1A, and TRAP1 genes in CRC. **(A)** Expression of SUCLG2, ACACB, OSBPL1A, and TRAP1 genes in the GSE20916 dataset. **(B)** Expression of SUCLG2, ACACB, OSBPL1A, and TRAP1 genes in the GSE21510 dataset.

The GEO database is used to analyze the gene expression of SUCLG2, ACACB, OSBPL1A, and TRAP1 in normal and cancerous colorectal tissues. Figure 5 shows that compared to normal tissues, the levels of ACACB and OSBPL1A increase in CRC tissues, while the levels of SUCLG2 and TRAP1 decrease in CRC tissues.

### 3.6 Association of risk score with clinicopathological factors

**FIGURE 6:**
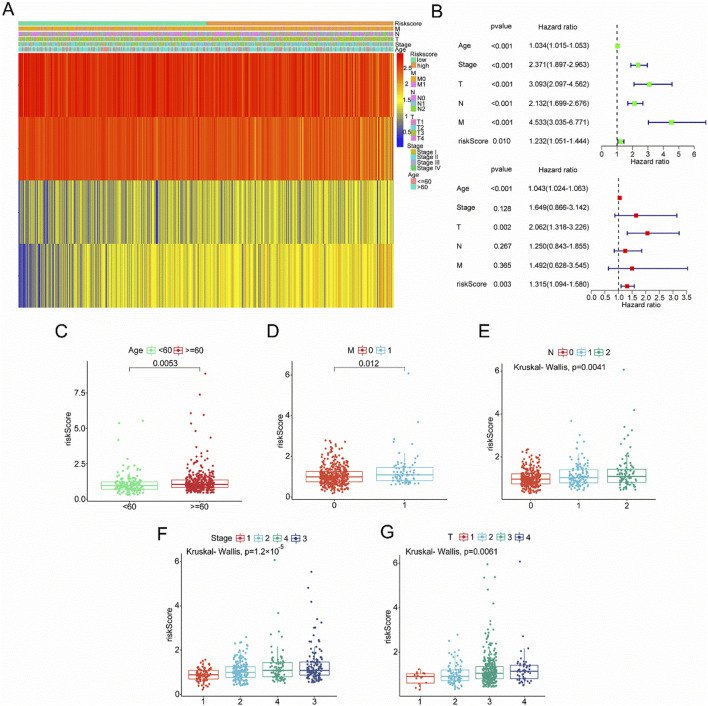
Association of Clinical Variables with Risk Score and Survival Analysis in Cancer Patients. **(A)** Heatmap of risk scores and various clinical characteristics (Age, Stage, T, N, M) in cancer patients. **(B)** Forest plot of hazard ratios and p-values from a Cox proportional hazards regression analysis for different clinical variables (Age, Stage, T, N, M) and risk score. **(C)** Age groups (below 60 and 60 or above) with a significant difference. **(D)** Metastasis status (0 vs. 1) showing a significant difference. **(E)** Lymph node involvement levels (0, 1, 2) showing a significant difference using the Kruskal–Wallis’s test. **(F)** Cancer stage (1, 2, 3, 4) with a significant difference in risk scores. **(G)** Tumor size categories (1, 2, 3, 4) showing a significant difference.

TCGA database results showed that higher risk scores correlate with increased expression of ACACB and OSBPL1A, while SUCLG2 and TRAP1 expression decreased (Figure 6A). Univariate and multivariate Cox analyses from TCGA revealed that age (p < 0.001), T stage (p = 0.002), and risk score were independent prognostic factors (Figure 6B). Significant differences were observed between the high-risk and low-risk groups regarding age, grade, and TNM stage (Figures 6C–G). Patients aged 60 and above have a higher risk of disease and a poorer prognosis (p = 0.0053). M1 stage patients have a worse prognosis compared to M0 (p = 0.012), and both N2 and N1 stages present a higher risk of disease than N0 (p = 0.0041). The disease risk progressively increases with advancing stage (p = 1.2 × 10^−5^). A similar trend is observed for T stages, where higher T stages are linked to greater disease risk (p = 0.0061).

### 3.7 Verify the expression of mitochondria-related features

**FIGURE 7:**
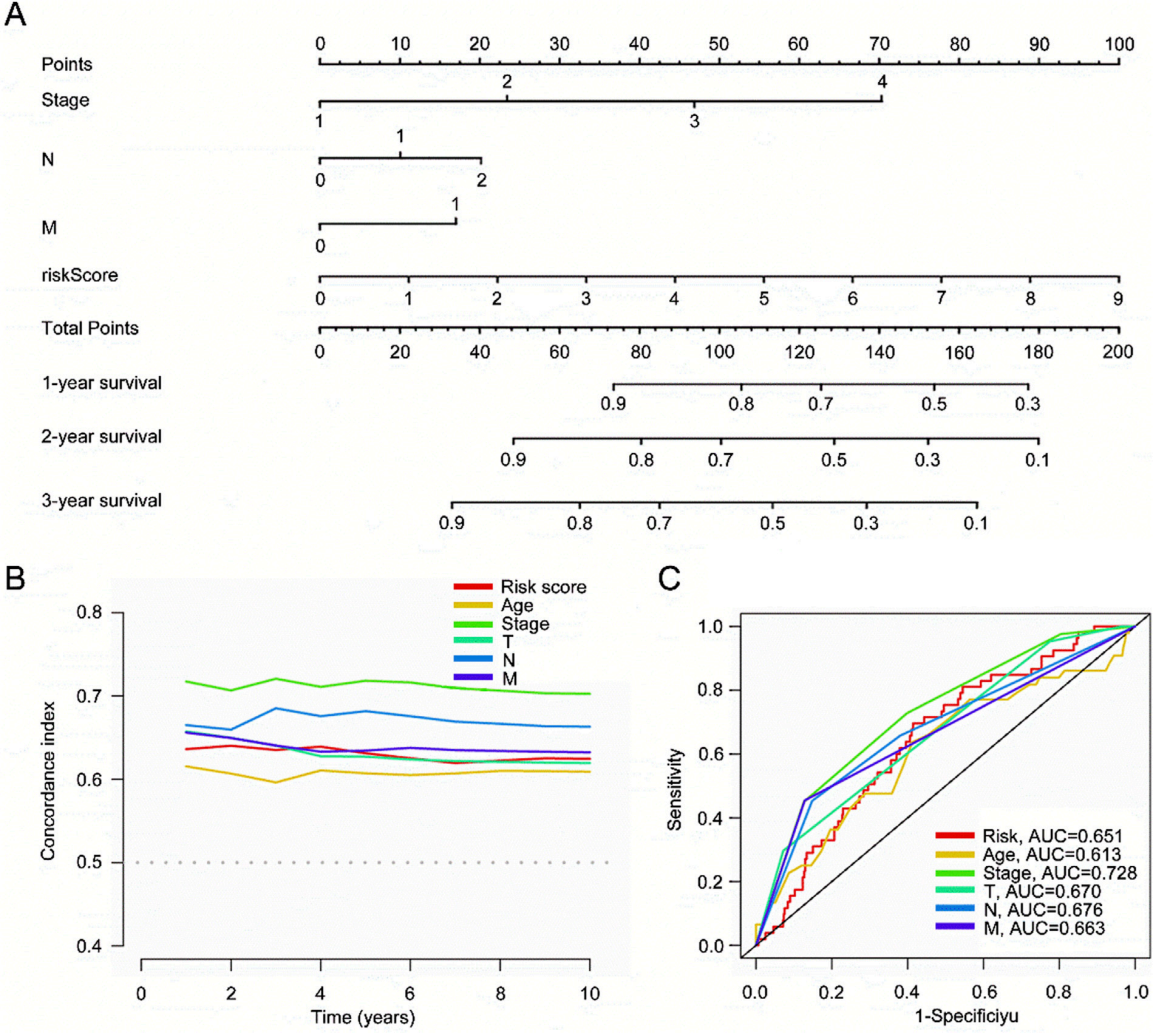
Construction of a nomogram to predict individualized survival probability for CRC patients. **(A)** Nomogram; **(B)** C-index of the nomogram; **(C)** ROC curves for clinical prediction of CRC risk scores.

Our nomogram gives a score to each prognostic indicator, and the total score is the sum of these individual scores (Figure 7A). The concordance index (C-index) shows that the nomogram aligns with the ideal model (Figure 7B). The ROC curves for clinical features and risk scores in the training set showed AUC values of 0.651 for the risk model, 0.613 for age, 0.728 for staging, 0.670 for T stage, 0.676 for N stage, and 0.663 for M stage (Figure 7C). The ROC analysis indicate that our nomogram is a reliable predictive model.

### 3.8 Mitochondria-related gene features are associated with immunity

**FIGURE 8:**
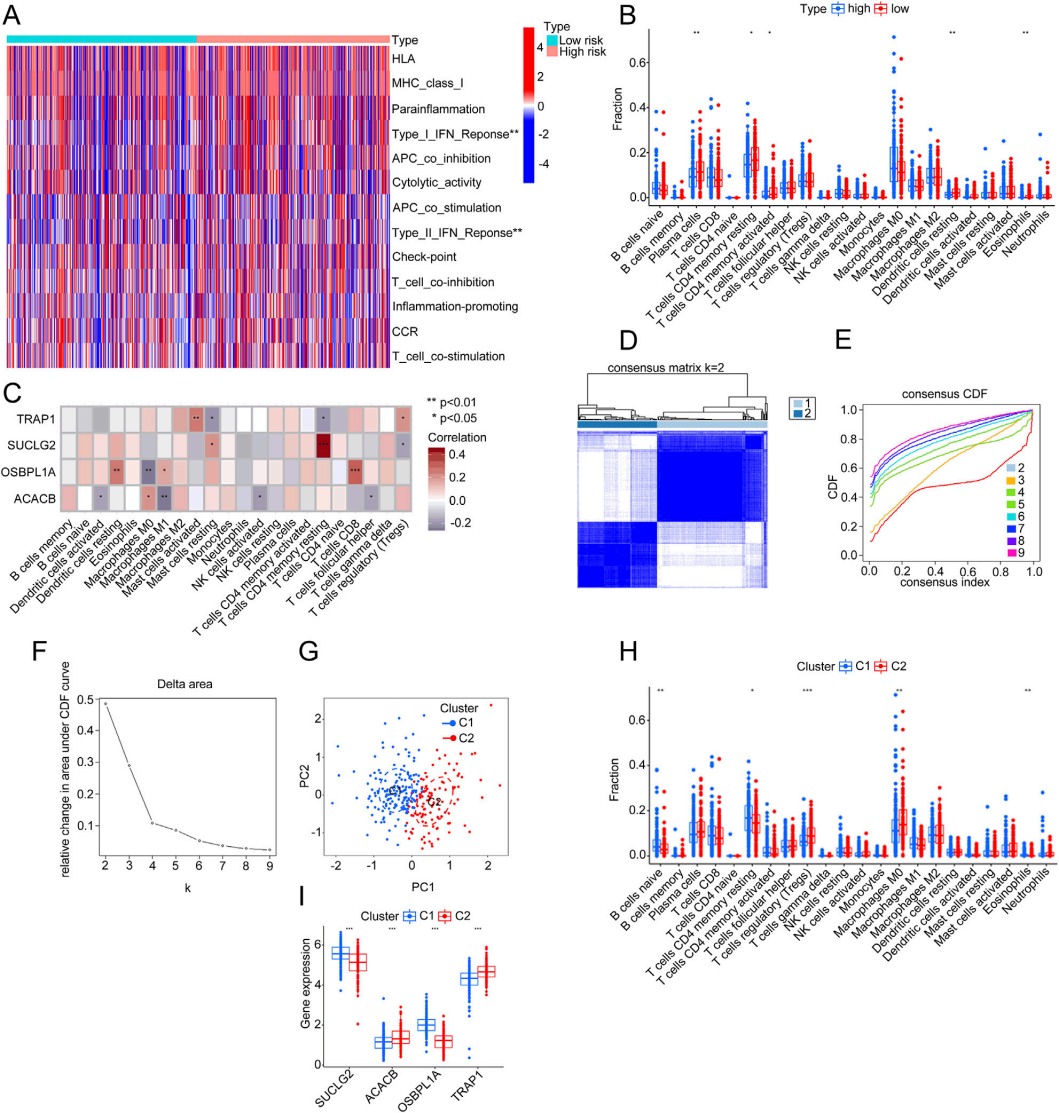
Differential analysis of immune characteristics and gene expression between risk and clustering groups. **(A)** Heatmap showing differences in the expression of various immune features between the high-risk and low-risk groups. **(B)** Comparative chart of immune cell infiltration proportions, contrasting the high-risk group (red) with the low-risk group (blue). **(C)** Correlation analysis of four genes (TRAP1, SUCLG2, OSBPL1A, and ACACB) with different types of immune cell infiltration. **(D)** Consensus matrix plot (k = 2) illustrating the clustering results. **(E)** Consensus cumulative distribution function (CDF) curve showing clustering stability across different k-values. **(F)** Delta area plot indicating relative changes in the CDF curve area at k = 2. **(G)** PCA plot illustrating the distribution of two clusters (C1 and C2) along PC1 and PC2. **(H)** Comparative chart of immune cell infiltration proportions between different clusters (C1 and C2). **(I)** Box plot showing expression levels of SUCLG2, ACACB, OSBPL1A, and TRAP1 genes across clusters (C1 and C2).

To analyze the relationship between risk groups and immune characteristics in CRC, we used an ssGSEA heatmap to display the expression differences across multiple immune-related features between high-risk and low-risk groups. Significant differences in immune features, specifically Type I and Type II IFN Responses, were observed between the groups (Figure 8A). A CIBERSORT bar chart further illustrated variations in immune cell infiltration proportions between high- and low-risk groups. And the results revealed that the notable differences in several immune cell types could activate CD4 memory T cells and resting dendritic cells (Figure 8B). The expression of SUCLG2, ACACB, OSBPL1A, and TRAP1 influenced these immune cell differences (Figure 8C, P < 0.05).

The consensus matrix identified two distinct clusters, suggesting that k = 2 yielded the best clustering results (Figure 8D). The consensus cumulative distribution function (CDF) curve confirmed the stability of clustering across various k-values. The smallest relative change occurred at k = 2, further indicating that k = 2 is the optimal choice for clustering (Figure 8E). The delta area plot illustrated the change in the CDF area as k-values increased, showing a significant decline at k = 2, reinforcing that k = 2 remains the optimal number of clusters (Figure 8F).

The PCA scatter plot showed the separation of two clusters, C1 and C2, along principal components 1 (PC1) and 2 (PC2), validating the clustering effectiveness (Figure 8G). A bar chart displayed the differences in immune cell infiltration proportions between the two clusters, C1 and C2, highlighting significant variations in types such as naive B cells, resting memory CD4 T cells, regulatory T cells, M0 macrophages, and eosinophils (Figure 8H). A box plot displayed the expression levels of SUCLG2, ACACB, OSBPL1A, and TRAP1 across the clusters (C1 and C2), with statistically significant differences in gene expression, suggesting their potential role in the immune microenvironment (Figure 8I).

### 3.9 A broad correlation between tumor mutation burden and stemness

**FIGURE 9:**
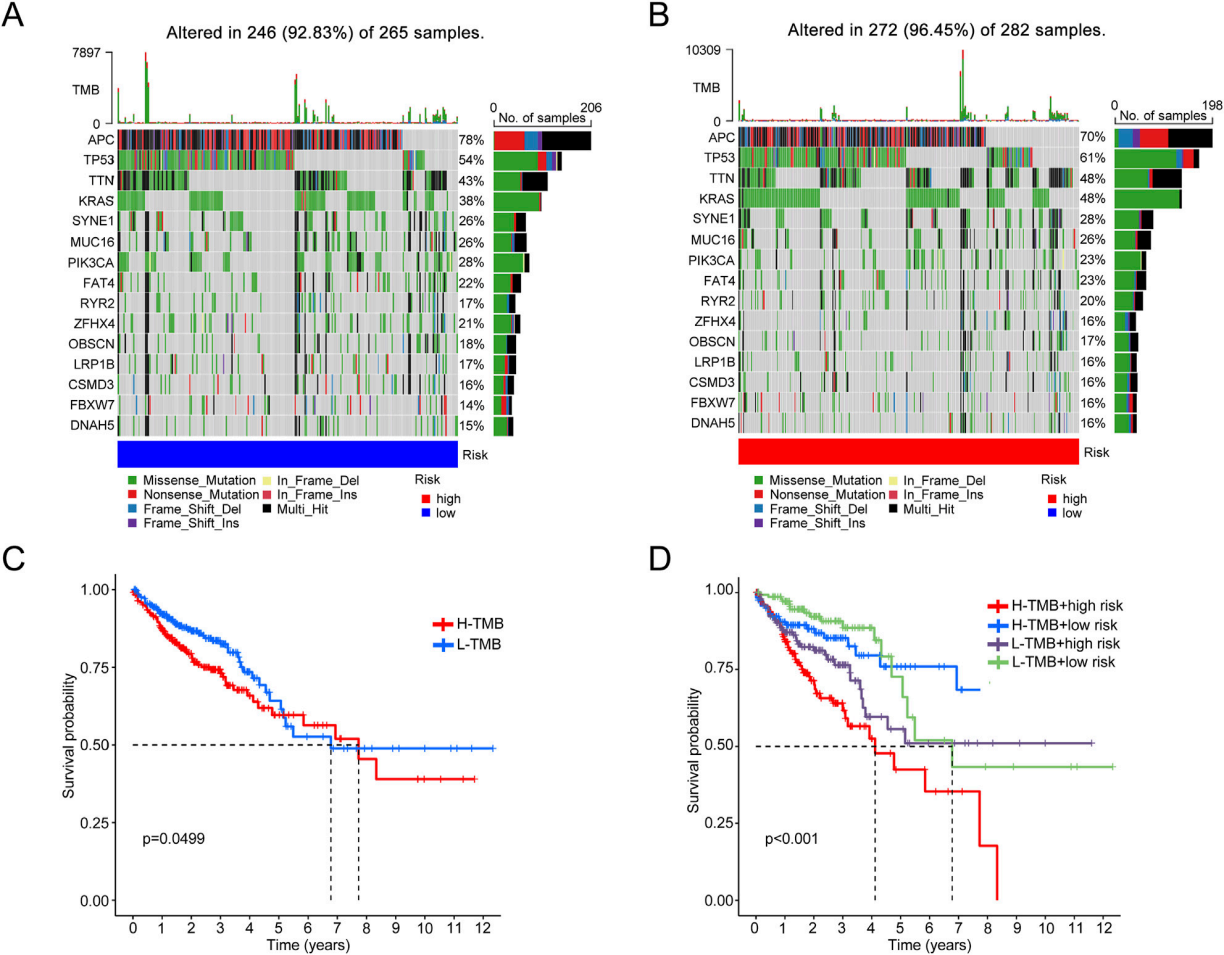
Analysis of gene mutation distribution and survival probability in different tumor mutation burden (TMB) levels and risk groups. **(A)** Waterfall plot of the gene mutation distribution in the common types and frequencies of gene mutations in the L-TMB group. **(B)** Waterfall plot of the common types and frequencies of gene mutations in the H-TMB group. **(C)** Survival curve comparing survival probabilities between the H-TMB and L-TMB groups. **(D)** Survival curve integrating the effects of TMB levels and risk groups.

The results show that the low-risk group has a lower percentage of mutations in various genes compared to the high-risk score group, which has higher mutation frequencies in genes like TP53 (61%) and KRAS (48%) (Figures 9A, B). Survival analysis shows a significant difference in survival rates between patients classified into high- and low-mutation burden groups (Figures 9C, D). These findings suggested a correlation between mutation burden, CRC, and mitochondrial function.

### 3.10 Drug sensitivity analysis and molecular docking

**FIGURE 10:**
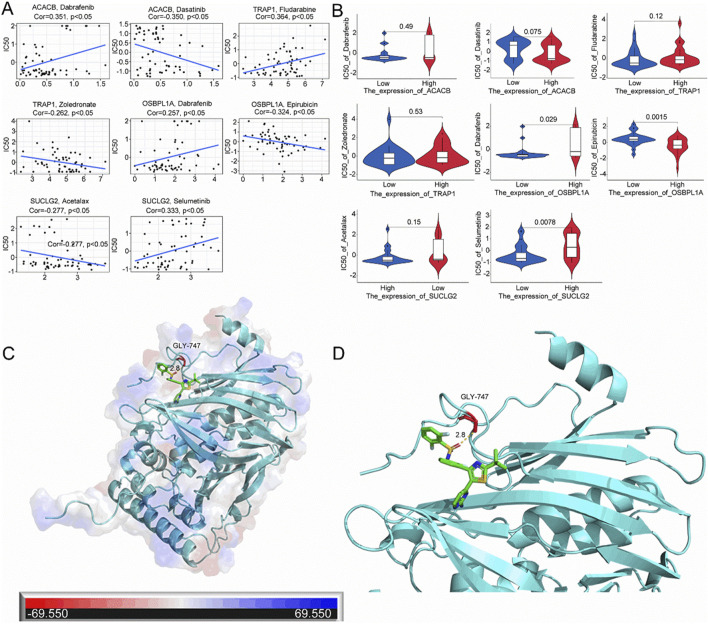
Sensitivity analysis of mitochondrial-associated prognostic genes and molecular targeted drugs. **(A)** Drug sensitivity correlation analysis for SUCLG2, ACACB, OSBPL1A, and TRAP1 using the CellMiner database; **(B)** Correlation analysis between the expression of SUCLG2, ACACB, OSBPL1A, and TRAP1 and corresponding drug sensitivities in the CellMiner database; **(C)** Molecular docking results between OSBPL1A and Dabrafenib; **(D)** Detailed view of the local docking interface between OSBPL1A and Dabrafenib.

A drug sensitivity analysis was performed to create treatments for all CRC patients. The results show a positive correlation between ACACB gene expression and sensitivity to Dabrafenib, while a negative correlation is observed with Dasatinib sensitivity. TRAP1 gene expression positively correlates with Fludarabine sensitivity but negatively correlates with Zoledronate sensitivity. OSBPL1A gene expression shows a positive correlation with Dabrafenib sensitivity and a negative correlation with Epirubicin sensitivity. SUCLG2 expression is negatively correlated with sensitivity to Acetalax and positively correlated with sensitivity to Selumetinib (Figure 10A). The correlation analysis of SUCLG2, ACACB, OSBPL1A, and TRAP1 gene expressions with their corresponding drug sensitivities revealed distinct patterns: OSBPL1A expression affects both Dabrafenib and Epirubicin sensitivities, while Selumetinib sensitivity is influenced by SUCLG2 expression (Figure 10B). Given that OSBPL1A is the gene with the highest disease risk (Figures 3A, D), and Dabrafenib exhibits a positive correlation with OSBPL1A sensitivity, OSBPL1A and Dabrafenib were selected for further analysis. Molecular docking between OSBPL1A and Dabrafenib indicates that this protein can bind through residues with a static potential energy of approximately 69,550 (Figure 10C). A close-up of the docking site reveals that Dabrafenib covalently binds to OSBPL1A at position 747 through a glycine residue (Figure 10D).

### 3.11 Dabrafenib inhibits the growth of HCT116 and SW480 cells

**FIGURE 11:**
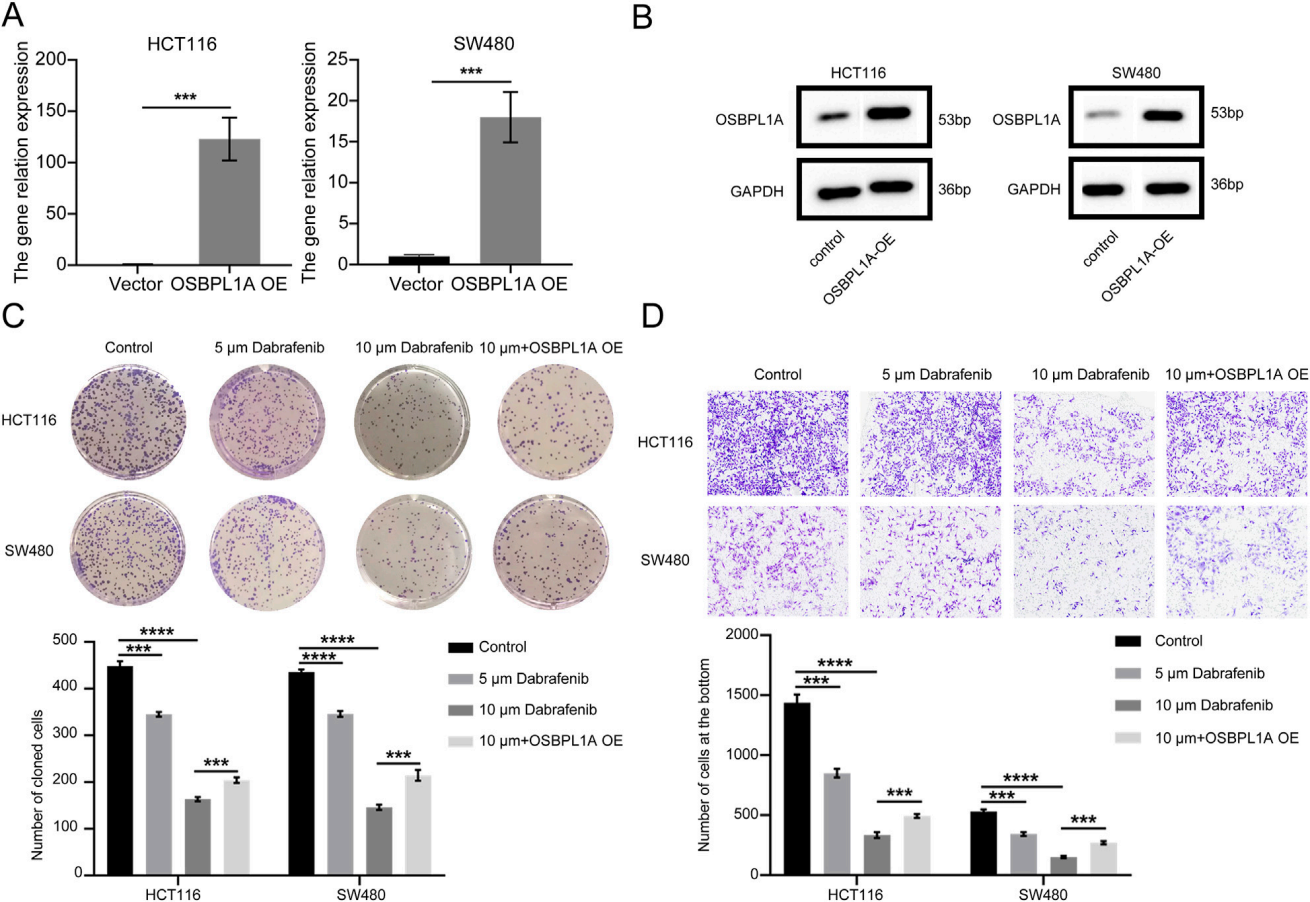
Evaluation of Dabrafenib’s potential as a treatment for CRC. **(A)** Efficiency of OSBPL1A overexpression in CRC cells, assessed by RT-qPCR analysis. **(B)** Evaluate the efficiency of OSBPL1A protein overexpression in colorectal cancer HCT116 and SW480 cells through Western blotting analysis. **(C)** Plate colony formation assay showing the effect of Dabrafenib on CRC cell proliferation. **(D)** Cell migration assay demonstrating the impact of Dabrafenib on the migratory ability of CRC cells.

Colony formation assays were conducted to evaluate whether Dabrafenib inhibits the proliferation of CRC cells, specifically HCT116 and SW480. The results indicated that the colony formation rate for both HCT116 and SW480 cells decreased as Dabrafenib concentrations increased. The colony formation rate of HCT116 and SW480 cells treated with 5 μM and 10 μM Dabrafenib was significantly lower than that of the control group (Figure 11C). Simultaneous OSBPL1A overexpression led to a 121.9-fold increase in HCT116 cell proliferation and a 16.99-fold increase in SW480 cell proliferation (Figure 11A). The protein level of OSBPL1A was also sgnificantly increased in both HCT116 and SW480 cells (Figure 11B). After OSBPL1A overexpression, both HCT116 and SW480 cells were treated with 10 μM Dabrafenib, and the colony formation rate was partially restored (Figure 11C). Transwell migration assay further showed that the migration rate of HCT116 and SW480 cells decreased with increasing Dabrafenib concentrations. Compared to the control group, fewer HCT116 and SW480 cells migrating to the lower chamber was significantly reduced after exposure to 5 μM and 10 μM Dabrafenib. However, after OSBPL1A overexpression, the number of migrated HCT116 and SW480 cells was partially restored (Figure 11D).

## 4 Discussion

In this study, we systematically analyzed mitochondrial-associated gene expression in CRC tissues and their prognostic implications. Our study revealed the potential mechanisms through which mitochondrial genes influence CRC progression and immune responses. Mit-DEGs play a crucial role in energy metabolism and oxidative processes, particularly in fatty acid oxidation and organic acid metabolism. These findings suggest that mitochondria are not only essential for maintaining cellular energy supply but may also impact tumor growth and metastasis by regulating the cellular redox state. Furthermore, Mit-DEGs are closely associated with the immune microenvironment of CRC, particularly in the observed differences in Type I and Type II interferon responses. Through ssGSEA and CIBERSORT analysis, we identified significant differences in immune cell infiltration between high-risk and low-risk groups, further emphasizing the pivotal role of mitochondrial genes in modulating the immune microenvironment. Additionally, we confirmed that the key Mit-DEGs are linked to a lower mutation frequency in the low-risk group, while the high-risk group is strongly associated with mutations in genes such as TP53 and KRAS. This suggests that mitochondrial function may influence CRC progression by altering the mutation burden in tumor cells.

We identified four mitochondrial-related genes and constructed a novel prognostic model for CRC. This mitochondrial-based prognostic nomogram can help manage CRC patients by guiding treatment decisions. Additionally, mitochondrial characteristics are independent prognostic predictors with higher sensitivity and specificity compared to traditional clinical features. We further compared immune features across different risk groups and analyzed the correlation between these mitochondrial genes and immune cells. Notably, we found a close relationship between mitochondrial-associated genes in CRC and tumor-infiltrating immune cells, including plasma cells, resting CD4 memory T cells, activated CD4 memory T cells, resting dendritic cells, and eosinophils. This finding provides new insights into the role of mitochondria in CRC immunotherapy. Despite extensive research on the mitochondrial aspects of tumors, mitochondrial markers are rarely used in prognostic models.

In recent years, mitochondria have played a crucial role in the development and progression of CRC. For instance, cancer cells that lack mitochondrial DNA (mtDNA) lose their tumor-forming ability. They can regain this potential only by acquiring mitochondria from the surrounding stroma, which allows them to perform oxidative phosphorylation (Valero, 2014). CRC cells exhibit significantly increased mitochondrial metabolic activity and produce higher levels of ROS. These cells require a greater glucose supply to meet their energy demands and display higher anabolic activity than normal cells (Tabe et al., 2019). Moreover, the shift toward anaerobic glycolysis enhances cancer cell proliferation by triggering hypoxia-induced activation (Chen et al., 2023). Toadstatin induces apoptosis in U251 cells by disrupting the mitochondrial fission/fusion homeostasis through the demyelination of mitochondrial outer membrane-associated protein A2 and DRP1 (Zuo et al., 2024; Lee et al., 2024). One of the most notable characteristics of cancer cells is their ability to evade apoptosis. Pro-carcinogenic alterations in the MEK/ERK signaling pathway induce the phosphorylation of Mfn-1, thereby inhibiting apoptosis (Pyakurel et al., 2015). These findings suggest that mitochondria play a crucial role in tumor and may be involved in the initiation and progression of CRC. However, the specific functions and molecular mechanisms of these mitochondrial-associated genes require further experimental investigation.

Immune activation is crucial for fighting cancer growth, while mitochondrial dynamics can affect cancer progression by influencing immune system activity, especially concerning T cells (Wang et al., 2023). Our analysis showed significant correlations between T cells, including CD8^+^ T cells, Th1 cells, and Th2 cells, in high-risk groups. Recent studies on peptide immunology, including IFN-γ ELISPOT assays, showed that FMACSPVAL effectively triggered Sox11-specific CD8^+^ T cells (Liu et al., 2023). This novel peptide epitope holds promise as a potential target for T cell-based immunotherapy in CRC. Conversely, another study indicated that M1 macrophages might promote tumorigenesis by modifying the tumor microenvironment, akin to the Warburg effect seen in cancer cells (Jaworska et al., 2023). Our findings also indicated that macrophage infiltration was notably higher in the high-risk group, potentially contributing to the poor prognosis of these patients. A study examining immune cell infiltration and immunotherapy in low-grade CRCs found that elevated immune infiltration scores were strongly associated with an increase in CD4 naïve T cell infiltration, with high CD4 naïve T cell expression correlating with a favorable patient prognosis (Wu et al., 2023). Furthermore, alterations in somatic mtDNA, or a reduced mtDNA copy number, are linked to enhanced cancer progression and metastasis by activating retrograde mitochondrial signaling pathways (Wang et al., 2023). Conversely, eliminating mtDNA has been shown to restrict tumor development. These results indicate that CRC can activate immune responses via CD4 T cells, CD8 T cells, and macrophages. Additionally, it can modulate metabolic processes by decreasing mtDNA content and increasing glycolysis, potentially providing a new strategy for effective CRC treatment.

CRC Patients who receive radiotherapy often experience high recurrence rates because cancer cells are resistant to radiation (McCarthy et al., 2012). There is an urgent need for therapies that target the mechanisms of radiotherapy resistance to enhance the radiation response and ultimately improve patient survival. A study revealed that in radiation-resistant CRC cells, the NADH ubiquinone oxidoreductase (complex I) subunit was significantly upregulated in the mitochondria. Additionally, there was an increase in mitochondrial DNA copy number. The study demonstrated that treating these resistant cells with mitochondrial complex I inhibitors resensitized the drug-resistant CRC cells to radiation (Zhu et al., 2023). Tumor hypoxia and altered metabolic states drive the malignant progression and drug resistance of cancer cells. Moreover, CRC stem cells show a preferential dependence on mitochondrial metabolism, suggesting that therapies targeting mitochondria could be promising for treating CRC stem cells (Sessions and Kashatus, 2021). An analysis of drug sensitivity indicated that the expression of mitochondrial genes OSBPL1A and ACACB was positively correlated with Dabrafenib. Mitochondrial proteins OSBPL1A and ACACB can enhance radiosensitivity, reduce drug resistance, and offer new hope for treating recurrent cancer. However, further clinical trials are needed to validate these findings.

OSBPL1A is a protein that plays a role in the transport and metabolism of lipids within cells. It is part of the oxysterol-binding protein family (Zhao et al., 2020). OSBPL1A regulates the distribution of cholesterol and phospholipids. It interacts with cellular membranes and other intracellular structures, which helps maintain membrane homeostasis and balance (Wyles and Ridgway, 2004). Mutations in OSBPL1A are associated with lipid metabolism disorders and may alter membrane structure, contributing to various metabolic diseases. Research on OSBPL1A in cancer is limited, and its role in tumors remains unclear. Our findings indicate that increasing OSBPL1A expression in CRC enhances its proliferation and migration abilities. This suggests that OSBPL1A and its associated signaling pathways may serve as potential therapeutic targets for anti-tumor strategies. These findings highlight the crucial role of OSBPL1A in tumorigenesis and lipid metabolism regulation.

Dabrafenib is a BRAF inhibitor primarily used to treat cancer with the BRAF V600E mutation. In CRC, approximately 8%–10% of patients harbor BRAF V600E mutations, which typically are associated with poor prognosis (Grothey et al., 2021). For patients with BRAF V600E mutant CRC, combination therapy with PD-1, BRAF, and MEK inhibitors significantly increased the clinical objective response rate (cORR) to 25% (95% CI 10.7%–44.9%). This rate is more than three times higher than the 7% (95% CI 1.5%–19.1%) observed in patients who had not been previously treated with BRAF inhibitors. In terms of sustained efficacy, the median progression free survival (PFS) of combination therapy is 5 months, compared to the 3.5 months of BRAF/MEK inhibition therapy alone. 57% of patients receive treatment for more than 6 months, and 18% of patients continue treatment for over 1 year (Tian et al., 2023). These data indicate that the therapeutic effect of Dalafenib combined with PD-1 and MEK inhibition in BRAF V600E mutant CRC patients deserves attention. In experiments testing the anticancer activity of Dabrafenib in CRC cells, plate clone formation assays showed that Dabrafenib significantly reduced the viability of CRC cell lines in a dose-dependent manner. In addition, Transwell experiments showed that 2.5 μM and 5 μM Dabrafenib inhibited the migration ability of CRC cells. These findings indicated that Dabrafenib could serve as an effective clinical therapy for CRC, providing new treatment options and personalized strategies for patients.

The study has several limitations. First, the data came from a database, and the findings need further validation in clinical settings to confirm the reliability of predictions regarding mitochondria-associated gene expression in CRC. Second, the impact of mitochondrial alterations on the immunophenotype of CRC has not been explored. This will be the focus of our future research to better understand how mitochondrial genes contribute to immune evasion in CRC. Additionally, the proposed drugs aimed at enhancing sensitivity need further validation through laboratory experiments and clinical trials.

## 5 Conclusion

This study examined mitochondrial characteristics in all stages of CRC and their potential as prognostic markers. The study identified three mitochondrial-related biological functions and key signaling pathways. These findings offer valuable insights into the molecular mechanisms that drive colorectal cancer progression. Moreover, the study developed a new prognostic signature and identified potential therapeutic agents tailored to individual patient profiles. These findings will enhance diagnostic and treatment strategies for CRC, offering a new approach for targeting mitochondrial function and integrating immunotherapy into clinical practice.

## Data Availability

The datasets presented in this study can be found in online repositories. The names of the repository/repositories and accession number(s) can be found in the article/Supplementary Material.
