# Supplementary material for: New prognostic features and personalized treatment strategies of mitochondrial related genes in colorectal cancer patients
Source: Front Pharmacol. 2025 Apr 1;16:1540767. doi: 10.3389/fphar.2025.1540767 (PMC12023264; doi:10.3389/fphar.2025.1540767)
Supplement: Supplementary file 1 [file DataSheet1.docx]

| Supplementary Table 1: Clinical information of CRC patients | | | |  |
| --- | --- | --- | --- | --- |
| Characteristics | COAD | | READ | |
|  | Number of cases | Percentages | Number of cases | Percentages |
| **OS** |  |  |  |  |
| Alive | 352 | 78.0 | 133 | 83.1 |
| Dead | 99 | 22.0 | 27 | 16.9 |
| **Age** |  |  |  |  |
| <65 | 172 | 38.1 | 75 | 46.9 |
| >=65 | 279 | 61.9 | 85 | 53.1 |
| **Gender** |  |  |  |  |
| Female | 208 | 46.1 | 74 | 46.3 |
| Male | 243 | 53.9 | 86 | 53.8 |
| **pT** |  |  |  |  |
| T1 | 12 | 2.7 | 7 | 4.4 |
| T2 | 79 | 17.5 | 28 | 17.6 |
| T3 | 308 | 68.3 | 111 | 69.8 |
| T4 | 52 | 11.5 | 13 | 8.2 |
| **pN** |  |  |  |  |
| N0 | 266 | 59.0 | 80 | 51.0 |
| N1 | 105 | 23.3 | 43 | 27.4 |
| N2 | 80 | 17.7 | 34 | 21.7 |
| N3 | 0 | 0.0 | 0 | 0.0 |
| **Metastasis** |  |  |  |  |
| No | 335 | 74.3 | 123 | 85.4 |
| Yes | 116 | 25.7 | 21 | 14.6 |
| **TNM** |  |  |  |  |
| I | 78 | 17.3 | 28 | 18.5 |
| II | 176 | 39.0 | 49 | 32.5 |
| III | 134 | 29.7 | 52 | 34.4 |
| IV | 63 | 14.0 | 22 | 14.6 |
|  |  |  |  |  |


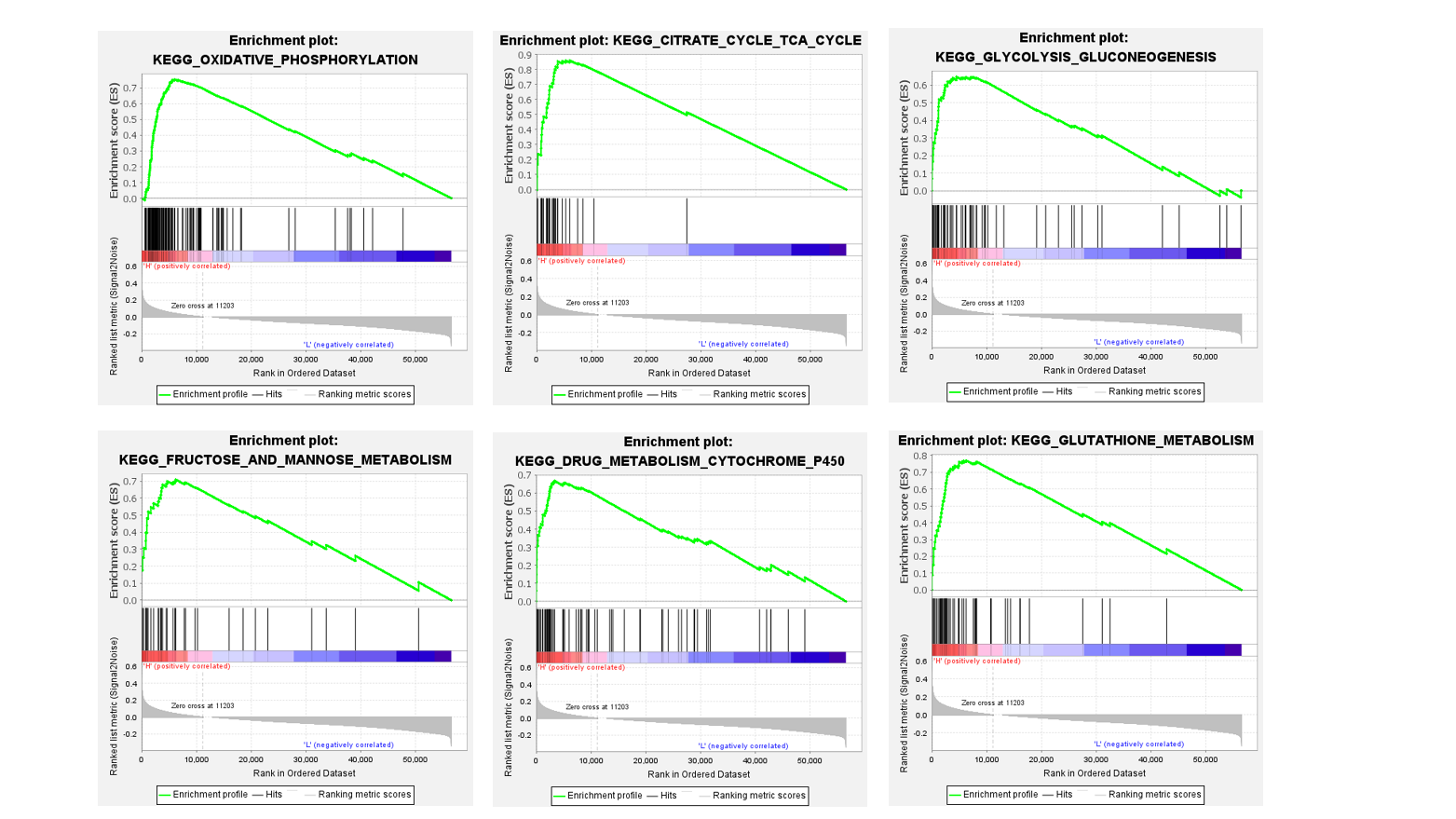


SUPPLEMENTARY FIGURE 1. The GSEA enrichment analysis identified relevant pathways through which Mit-DEGs regulate mitochondrial function.
